# Sirt4 Modulates Oxidative Metabolism and Sensitivity to Rapamycin Through Species-Dependent Phenotypes in *Drosophila* mtDNA Haplotypes

**DOI:** 10.1534/g3.120.401174

**Published:** 2020-03-09

**Authors:** Richard Sejour, Roger A. Sanguino, Monika Mikolajczak, Walishah Ahmadi, Eugenia Villa-Cuesta

**Affiliations:** * Biology Department, Adelphi University, Garden City, NY; † NYU Winthrop Research Institute, Mineola, NY

**Keywords:** *Sirt4*, TOR pathway, coevolution mtDNA/nDNA

## Abstract

The endosymbiotic theory proposes that eukaryotes evolved from the symbiotic relationship between anaerobic (host) and aerobic prokaryotes. Through iterative genetic transfers, the mitochondrial and nuclear genomes coevolved, establishing the mitochondria as the hub of oxidative metabolism. To study this coevolution, we disrupt mitochondrial-nuclear epistatic interactions by using strains that have mitochondrial DNA (mtDNA) and nuclear DNA (nDNA) from evolutionarily divergent species. We undertake a multifaceted approach generating introgressed *Drosophila* strains containing *D. simulans* mtDNA and *D. melanogaster* nDNA with *Sirtuin 4* (*Sirt4**)*-knockouts. Sirt4 is a nuclear-encoded enzyme that functions, exclusively within the mitochondria, as a master regulator of oxidative metabolism. We exposed flies to the drug rapamycin in order to eliminate TOR signaling, thereby compromising the cytoplasmic crosstalk between the mitochondria and nucleus. Our results indicate that *D. simulans* and *D. melanogaster* mtDNA haplotypes display opposite Sirt4-mediated phenotypes in the regulation of whole-fly oxygen consumption. Moreover, our data reflect that the deletion of *Sirt4* rescued the metabolic response to rapamycin among the introgressed strains. We propose that Sirt4 is a suitable candidate for studying the properties of mitochondrial-nuclear epistasis in modulating mitochondrial metabolism.

The endosymbiotic theory presents a logical framework for the evolutionary origins of mitochondria. Under this theory, proto-mitochondria capable of aerobic respiration entered a proto-eukaryote (McFadden 2001; Gatti 2016). However, rather than the proto-eukaryote (host) expunging, or succumbing to the proto-mitochondria’s (endosymbiont) virulence, a mutualistic relationship ensued wherein the endosymbiont specialized in producing the majority of the cell’s energy through aerobic respiration, whereas the host provided a stable environment rich with metabolic substrates (Archibald 2015). The endosymbiont assimilated into the host through retrograde (mitochondria to nucleus) genetic transfers and subsequent nuclear integration of mitochondrial genes vital for mitochondrial survival and function (Timmis *et al.* 2004) causing the progressive truncation of the mitochondrial genome (Kleine *et al.* 2009). This ongoing process is referred to as the coevolution between the nuclear and mitochondrial genomes, which serves as the foundation in the crosstalk between the nucleus and mitochondria that is essential for metabolic homeostasis (Guaragnella *et al.* 2018).

Effective communication between mitochondria and other organelles is facilitated through cytoplasmic pathways that integrate signaling in response to shifts in the availability of cellular nutrients and stress (Butow and Avadhani 2004). Among these cytosolic pathways is the target of rapamycin (TOR). TOR is an evolutionarily conserved serine-threonine kinase that mediates anterograde (nuclear to mitochondria) and retrograde (mitochondria to nuclear) communication through signal transductions concomitant with metabolism, cell survival, proliferation, and anti-apoptosis (Dowling *et al.* 2010). The prolific cellular processes associated with TOR signaling are largely coordinated by two major complexes: TORC1 and TORC2. Dysregulated TOR activity has been associated with abnormal metabolic functions, diabetes, as well as cancers such as melanoma, glioblastoma, and renal cell carcinoma (Zoncu *et al.* 2011; Pópulo *et al.* 2012; Wu *et al.* 2014). The most studied TOR inhibitor is rapamycin. Rapamycin is an FDA-approved macrolide with potent immunosuppressant and anti-tumorigenic properties (Gaumann *et al.* 2008), that is known to inhibit TORC1 through competitive allosteric interactions with the FKBP12-rapamycin binding (FRB) (Vilella-Bach *et al.* 1999).

One of the ways that TOR modifies mitochondrial metabolism is through activation of glutamate dehydrogenase (GDH). This activation requires the repression of Sirt4, an enzyme that inhibits GDH (Csibi *et al.* 2013). Sirt4 belongs to an evolutionarily conserved family of nicotinamide adenine dinucleotide (NAD+)-dependent deacetylases that are important in regulating cellular processes (Yamamoto *et al.* 2007). Although the Sirt4 enzyme is localized within the mitochondria, the *Sirt4* gene is encoded within the nuclear genome (van de Ven *et al.* 2017). Mammalian Sirt4 has been well characterized as a chief antagonist of oxidative metabolism by interfering with key anaplerotic processes of the Krebs cycle. Sirt4 incorporates NAD+ as a cofactor and source of ADP-ribose (Haigis *et al.* 2006), thus establishing Sirt4 in the negative feedback regulation on metabolism during states when mitochondrial NAD+ levels are high, most notably during oxidative phosphorylation. As the gatekeeper of metabolism, Sirt4 exhibits the following enzymatic properties in mammals: reducing fatty acid oxidation in mice (Nasrin *et al.* 2010), the inhibition of pyruvate dehydrogenase through its catalytic role as a lipoamidase (Mathias *et al.* 2014), and the inhibition of glutamate dehydrogenase through mono ADP-ribosyltransferase activity (Haigis *et al.* 2006). Conversely, recent studies reveal different Sirt4 phenotypes in fruit flies as indicated by *Sirt4*-knockout *D. melanogaster* retaining higher levels of energy reserves in the form of branched-chain amino acids (valine, leucine and isoleucine), fatty acids, and glycolytic metabolites (Wood *et al.* 2018). Under starvation, the capacity for *Sirt4*-knockout flies to metabolize and convert energy stores is greatly compromised (Wood *et al.* 2018). As a whole, it is apparent that Sirt4 exerts opposite metabolic phenotypes in mammals and *Drosophila*, although there is little research to explain the evolutionarily divergent mechanisms of Sirt4-dependent pathways.

In this research, we introgressed *Drosophila* mitochondrial DNA (mtDNA) and nuclear DNA (nDNA) from evolutionarily divergent species (*D. melanogaster* and *D. simulans*) in order to disrupt the coevolution between *Drosophila* nuclear and mitochondrial genomes (Davis *et al.* 1996; Montooth *et al.* 2010). Although the introgressed *Drosophila* strains do not exhibit profound incompatibilities between the mitochondrial and nuclear genomes (Montooth *et al.* 2010), they are insensitive to the effects of rapamycin on mitochondrial metabolism (Villa-Cuesta *et al.* 2014). Conversely, rapamycin-fed wildtype *D. melanogaster* exhibit trends of enhanced mitochondrial efficiency including: reduced levels of ketone bodies, upregulation of carnitine, and shifts of metabolism conferring upregulated amino acid and fatty acid catabolism (Villa-Cuesta *et al.* 2014). In the absence of rapamycin, the metabolic profile of introgressed lines reflected basal differences in mitochondrial metabolites when compared to wildtype *D. melanogaster* (Villa-Cuesta *et al.* 2014). These differences may be due to the non-optimal genetic epistasis between *D. melanogaster* nDNA and *D. simulans* mtDNA.

To better elucidate the role of key players in the nuclear-mitochondrial epistasis that secure functional crosstalk among cellular compartments, in this research we measure the oxygen consumption of introgressed strains in the presence of the TOR inhibitor rapamycin. Oxygen consumption served as a proxy for routine-fly metabolic rate; this metric abolishes artifacts generated by the *in vitro* manipulation and isolation of mitochondria from its cellular context while also providing a metabolic phenotype that characterizes mitochondrial-cellular communication (Lighton 2008). Given that the mitochondria produces the majority of the cell’s energy demands through oxidative phosphorylation, we hypothesize that disruption of the crosstalk, among the mitochondria and other cellular compartments, will result in changes in mitochondrial metabolism as well as whole-fly metabolic rate.

Our research shows that rapamycin elicits species-dependent metabolic phenotypes that are distinctly unique between *D. simulans* and *D. melanogaster*. Additionally, our data suggests that disrupting the coevolution between the nuclear and mitochondrial genomes alters metabolism, as characterized by Sirt4 conferring resistance to rapamycin among introgressed strains. Our findings provide strong evidence that Sirt4’s metabolic phenotypes are governed by nuclear-mitochondrial epistatic interactions that mediate nutrient-sensing as well as energy conversion from bio-available metabolites.

## Materials and Methods

### Fly stocks and husbandry

**Figure 1:**
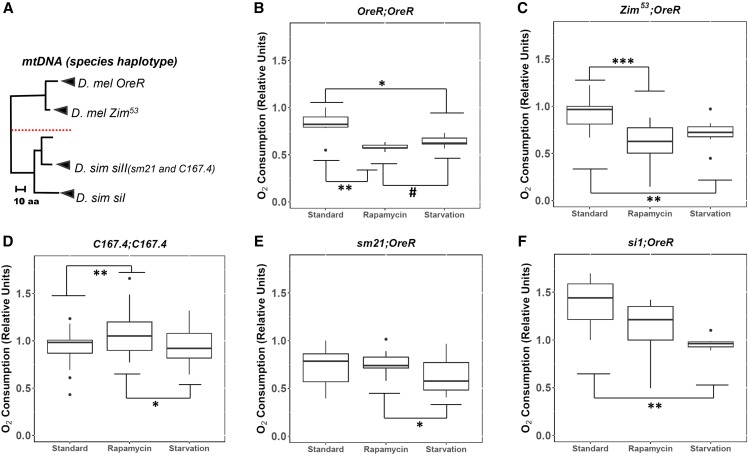
The effects of rapamycin on the whole-fly metabolic rate across *Drosophila* from divergent mitochondrial and nuclear lineages. (A) mtDNA phylogeny between the five mitochondrial haplotypes used in this experiment. The tree is grouped based on similarities in the composition of amino acid sequences. The red line demarcates evolutionarily divergent species. The name of the species is followed by the mitochondrial haplotype. In parentheses are the lines from which the mtDNA was isolated (modified and adapted from Montooth *et al.* 2010). Whole-fly oxygen consumption was measured in coevolved *D. melanogaster* mtDNA and nDNA: (B) *OreR;OreR* and (C) *Zim^53^;OreR*, *D. simulans* mtDNA and nDNA: (D) *C167.4;C167.4*, and the introgressed lines harboring *D. simulans* mtDNA in *D. melanogaster* nDNA background: (E) *sm21;OreR* and (F) *siI;OreR* after treatment with rapamycin and starvation. For each strain identifier, the left side represents the mitochondrial haplotype, and the right side represents the nuclear background. For each pairwise comparison, the raw p-values, Holm-Bonferroni corrected p-values, and Hedges’ g effect sizes are presented in Table 1 and File S2. # = 0.0.5 < *P* < 0.1; * = 0.01 < *P* < 0.05; ** = 0.001 < *P* < 0.01; *** = *P* < 0.001.

*OreR;OreR*, *Zim^53^;OreR*, *sm21;OreR*, and *si1;OreR Drosophila* strains were generated through controlled breeding between female *D. simulans* (*C167.4;C167.4* strain) and male *D. melanogaster* (*In(1)AB* strain*)* followed by iterative backcrossing of the female hybrids with *D. melanogaster* males (Davis *et al.* 1996) as described in Montooth *et al.* (2010). The genetic background of each strain consisted of nDNA from *OreR*, but the mtDNA haplotypes originated from evolutionarily divergent lineages (Figure 1A). More specifically, *OreR;OReR* and *Zim^53^;OreR* contained mtDNA from *D. melanogaster*, whereas the mtDNA of the introgressed strains (*sm21;OreR* and *si1;OreR*) were inherited from *D. simulans*. The BDSC_8840 stock was obtained from the Bloomington Drosophila Stock Center (https://bdsc.indiana.edu). BDSC_8840 are *Sirt4*-knockout mutants with the following genotype: w^1118^ TI{TI}Sirt4^white+1^; sna^Sco^/CyO, S^2^ (https://flybase.org/reports/FBgn0029783.html), in which *Sirt4* was completely ablated and replaced by the mini-*white* marker, *Sirt4white*^*+1*^, through ends-out recombination (Xie and Golic 2005; Gong and Golic 2003). BDSC_8840 was backcrossed with *w^1118^* for several generations to preserve the genetic background of the stocks. *OreR;OreR*, *Zim^53^;OreR*, *sm21;OreR*, and *si1;OreR* were crossed with BDSC_8840 in order to generate *Sirt4*-knockout mutants for each mitochondrial haplotype. In order to generate *Sirt4* wildtype strains, *OreR;OreR*, *Zim^53^;OreR*, *sm21;OreR*, and *si1;OreR* were individually crossed with *w^1118^* mutants. It should be noted that the metabolic and physiological trends of *w^1118^* were not statistically different when compared to the wildtype mtDNA haplotypes (data not shown). For each stock, the presence or absence of *Sirt4* was confirmed through PCR (Figure S1). An additional wildtype strain, *C167.4;C167.4* (coevolved mitochondrial and nuclear genomes from *D. simulans*), was included in this experiment. For each strain used in this experiment, males were backcrossed four times with females of the corresponding mtDNA haplotype in order to minimize genetic drift.

### Dietary conditions

The stocks were amplified in standard fly food: 0.79% agar, 2% SAF yeast, 5.2% cornmeal, 11% sucrose, and 1.125% Tegosept (20% methylparaben dissolved in ethanol). The stocks were maintained under stable conditions: 25°, 60% Relative Humidity, and 12 hr light/dark cycles. In the experimental treatment, rapamycin was dissolved in 190 proof ethanol and added to the standard food for a final concentration of 200 µM rapamycin as described in Villa-Cuesta *et al.* (2014). The experimental and vehicle treatments contained 0.38% ethanol. 30 female flies of the same age (one to two days old) were subject to the vehicle/standard food or 200 µM rapamycin for 10 days. In order to induce starvation, flies were transferred from standard fly food to a treatment of 1% agar in water 21 hr prior to the closed-flow respirometry assays. The flies were transferred into fresh food every three days. For each treatment, at least three biological replicates were established per sample. Each experiment (block) was repeated at least three times.

### Closed-flow respirometry

Following the 10 days of exposure to the dietary treatments, the routine metabolic rate of each sample was measured through closed-flow respirometry using a Field Metabolic System (FMS), which allows for accurate measurements of oxygen and carbon dioxide within an enclosed chamber. Flies were placed into a pre-weighed, 10 mL syringe and were weighed. Then, each syringe was flushed for 5 min with ambient air and the chamber was sealed to obstruct the passage of air. Each syringe of flies was considered a sample. Each sample was kept in the incubator for 60-80 min. Following the incubation period, 5 mL of each sample was injected into the FMS. Lastly, since some of the 30 flies may have died or escaped during this process, at the end of the respirometry analyses, the number of flies per sample was recorded and used to calculate the average rate of oxygen consumption per fly.

### Data analysis

Oxygen consumption was measured using Expedata-P Data Analysis Software designed by Sable Systems International, located in North Las Vegas, Nevada. The 5 mL samples were output as peaks, and the peaks were integrated against minutes; the area of the peaks quantified the rate of oxygen consumption and carbon dioxide emission. The O_2_ and CO_2_ measurements were lag corrected to account for the unequal retardation of molecules through the FMS (Lighton 2008). The volumetric oxygen consumption and volumetric carbon dioxide production (expressed as milliliters per minute) were calculated as described (Lighton 2008):

$$Vo_{2}=FR_{e}\left[\left(F_{i}o_{2}-F{’}_{e}o_{2}\right)-F{’}_{i}o_{2}*\left(F{’}_{e}co_{2}-F_{i}co_{2}\right)\right]/\left(1-F{’}_{i}o_{2}\right)$$

$$Vco_{2}=FR_{e}\left[\left(F{’}_{e}co_{2}-F_{i}co_{2}\right)+F_{i}co_{2}*\left(F_{i}o_{2}-F{’}_{e}o_{2}\right)\right]/\left(1+F_{i}co_{2}\right)$$

**Vo_2_** represents the rate of a sample’s oxygen consumption within a chamber, whereas **Vco_2_** represents the rate of carbon dioxide production; both were measured in milliliters per minute. The excurrent mass flow rate of gas species within the chamber, at STP (standard temperature and pressure), is denoted by **FR_e_**. The fractional concentration of oxygen entering the respirometer chamber (incurrent flow) is expressed as **F_i_o_2_**, which is equivalent to the fractional concentration of oxygen in ambient air (0.2094) assuming that CO_2_ and water vapor are scrubbed or otherwise eliminated from the system prior to data collection. **F’_e_o_2_** reflects the fractional concentration of oxygen leaving the chamber (excurrent flow) during the absence of water vapor. **F’_e_co_2_** symbolizes the excurrent fractional concentration of carbon dioxide without the presence of water vapor, whereas **F_i_co_2_** represents the incurrent fractional concentration of carbon dioxide. Since carbon dioxide was scrubbed from the system prior to data collection, the value of **F_i_co_2_** was zero.

After accounting for the experimental conditions, the simplified formulas are detailed below:

$$Vo_{2}=FR_{e}\left[\left(F_{i}o_{2}-F{’}_{e}o_{2}\right)-0.2094*\left(F{’}_{e}co_{2}\right)\right]/\left(1-0.2094\right)$$

$$Vco_{2}=FR_{e}\left[\left(F{’}_{e}co_{2}\right)\right]/1$$

In essence, **Vo_2_** and **Vco_2_** report shifts in the flow rate of oxygen and carbon dioxide within the syringe as a process of each sample’s cellular respiration throughout the incubation period. These calculations assume controlled settings in which the ambient air flushed into the syringe (before being sealed at the 10 mL mark) contained an oxygen fractional concentration of 0.2094 and a carbon dioxide fractional concentration of 0. After the incubation period, any changes in the gas concentrations can be attributed to the intrinsic differences in metabolism exhibited by each sample. Following data acquisition, the raw Vo_2_ were adjusted to account for the variation in the number of flies, incubation duration, as well as other transient conditions such as temperature.

### Approach to statistical analyses

For the measurements on *w^1118^* and *Sirt4white*^*+1*^, individual blocks consisting of *OreR;OreR* and a second strain (*siI;OreR*, *sm21;OreR*, or *Zim^53^;OreR*) were designated in a single experimental cycle. All values were normalized in standard units with respect to the first sample of *OreR w^1118^* under the standard treatment. For analysis of the wildtype strains (no manipulation of *Sirt4* or *white*), the data were normalized to the first sample of the strain in the standard treatment. It was necessary to normalize each block in this manner considering that the FMS is highly sensitive to fluctuations in external factors (relative humidity, gases, and temperature), and the experimental blocks were performed on different days (Lighton 2008). This approach further reduced sources of extrinsic variations, such as individualized handling methods, from influencing the metabolic rate of the samples. Each block was repeated at least three times.

Regression analyses revealed that non-normalized, whole-fly oxygen consumption (MRO2perhr) and the mass of the flies exhibited a moderate to high positive correlation (R = 0.57; *P* < 0.005) (Figure S2A). However, normalizing each sample’s oxygen consumption to the reference sample (*OreR w^1118^* in the standard treatment) within each block, abolished this linear relationship (R=-0.05 and *P* = 0.22) (Figure S2A). Moreover, exposure to rapamycin does not appear to significantly alter fly locomotive activity (*P* = 0.26 for wildtype *OreR;OreR*) (Figure S2B). No covariates were included since otherwise confounding variables were tightly controlled and factored into the analyses, such as the age of the flies, number of flies per sample, incubation duration, and the concentration of rapamycin.

The data from all the blocks were compiled and statistically analyzed using The R Project for Statistical Computing (R Core Team 2019). A 3-way ANOVA was conducted using oxygen consumption normalized to the reference sample as the dependent variable, and the following independent variables: treatment (rapamycin or ethanol vehicle), genotype (expression or deletion of *Sirt4*), and mtDNA (*D. melanogaster* or *D. simulans*). Regression diagnostics were performed on the model to identify influential values that could skew the statistical analyses. Influential values were identified as having a cook’s distance greater than twice the amount of the cutoff value of 4/(*N-K*-1), where *N* is the total number of observations and *K* is the number of explanatory variables (treatment, genotype, and mtDNA). In total, 4 out of 264 samples had respective cook’s distances that substantially skewed the model and were subsequently omitted from the analyses involving the *w^1118^* and *Sirt4white*^*+1*^ mutants. Similarly, influential data were omitted from the following analyses on the wildtype mtDNA haplotypes: *C167.4*;*C167.4* (3 out of 90), *OreR*;*OreR* (1 out of 18), *Zim53*;*OreR* (1 out of 36), *sm21*;*OreR* (1 out of 36), and *si1*;*OreR* (0 out of 18). For each pair-wise comparison independent *t*-tests were conducted at a significance level of 0.05; p-values were corrected using the Holm–Bonferroni method for analyses involving multiple comparisons. Additionally, effect sizes were computed to quantify the magnitude of effect that each condition had on oxygen consumption. Trends of oxygen consumption among each sample were visually displayed as boxplots to emphasize the degree of intrinsic variation and uniqueness in the metabolic response that each sample exhibited.

The standard deviation was pooled for all Hedges’ g estimates (Table 1). For each pair-wise analysis, ANOVA or Wilcoxon rank test were run depending on the normality (measured using the Shapiro-Wilk test of normality) and homoscedasticity (measured using Levene’s Test of Equality of Variances) of the model. The criteria for ANOVA were if the model satisfied requirements of both the Levene’s test (*P* > 0.1) and Shapiro-Wilk Normality Test (*P* > 0.05). If the data were normally distributed, but the groups/comparisons had unequal variances, then the HC4 estimator of OLS parameter estimates was used to adjust the model. The HC4 estimator is uniformly more robust than other estimators (HC0, HC1, HC2, and HC3) when dealing with heteroscedastic data containing high leverage values (Cribari-Neto 2004; Hayes and Cai 2007), which is common among *in vivo* experiments in *Drosophila*. If the data were profoundly non-normally distributed, then the nonparametric Wilcoxon rank-sum test was used regardless if the groups had equal or unequal variances. These statistics can be found in File S2.

**Table 1:** Parametric analyses were performed for each comparison in this table. P-values are presented as raw and corrected for multiple comparisons using the Holm-Bonferroni method. For effect sizes, Hedges’ g correction was applied at the 0.95 confidence level, and the variances were pooled for each pair-wise comparison. Regression diagnostics (sample sizes, normality, group variances, etc) can be accessed from File S2. The abbreviations represent the following: wt= wildtype mtDNA haplotypes outlined in Figure 1A, *w^1118^*= mtDNA haplotypes that express *Sirt4* but are knockouts of the *white* gene, *Sirt4^white+1^*= mtDNA haplotypes with the deletion of *Sirt4*. Std.= standard treatment, Rapa= rapamycin treatment, D. mel= *D. melanogaster*, D. sim= *D. simulans*

ID#					Raw p-value	Holm	Hedge G	Magnitude
1	Std.	OreR	*D. mel*	*w^1118^ vs. Sirt4^white+1^*	0.0374	0.448	0.517	medium
2	Std.	*Zim53*	*D. mel*	*w^1118^ vs. Sirt4^white+1^*	0.485	1	0.28	small
3	Std.	sm21	*D. sim*	*w^1118^ vs. Sirt4^white+1^*	0.36	1	−0.368	small
4	Std.	si1	*D. sim*	*w^1118^ vs. Sirt4^white+1^*	0.0407	0.448	−1.15	large
5	Rapa	OreR	*D. mel*	*w^1118^ vs. Sirt4^white+1^*	0.42	1	0.197	negligible
6	Rapa	*Zim53*	*D. mel*	*w^1118^ vs. Sirt4^white+1^*	0.0536	0.526	0.804	large
7	Rapa	sm21	*D. sim*	*w^1118^ vs. Sirt4^white+1^*	0.405	1	0.335	small
8	Rapa	si1	*D. sim*	*w^1118^ vs. Sirt4^white+1^*	0.84	1	0.0919	negligible
9	*w^1118^*	OreR	*D. mel*	Std. *vs.* Rapa	0.000018	0.000288	1.13	large
10	*w^1118^*	*Zim53*	*D. mel*	Std. *vs.* Rapa	0.284	1	0.432	small
11	*w^1118^*	sm21	*D. sim*	Std. *vs.* Rapa	0.405	1	0.335	small
12	*w^1118^*	si1	*D. sim*	Std. *vs.* Rapa	0.498	1	0.332	small
13	*Sirt4^white+1^*	OreR	*D. mel*	Std. *vs.* Rapa	0.00341	0.0512	0.74	medium
14	*Sirt4^white+1^*	*Zim53*	*D. mel*	Std. *vs.* Rapa	0.0526	0.526	0.808	large
15	*Sirt4^white+1^*	sm21	*D. sim*	Std. *vs.* Rapa	0.0237	0.307	0.958	large
16	*Sirt4^white+1^*	si1	*D. sim*	Std. *vs.* Rapa	0.00376	0.0526	1.65	large
17	wt	C167.4	*D. sim*	Std. *vs.* Rapa	0.00917	0.0275	−0.693	medium
18	wt	C167.4	*D. sim*	Std. *vs.* Starvation	0.866	0.866	−0.044	negligible
19	wt	C167.4	*D. sim*	Rapa *vs.* Starvation	0.0154	0.0309	0.653	medium
20	wt	*Zim53*	*D. mel*	Std. *vs.* Rapa	0.000669	0.00201	1.6	large
21	wt	*Zim53*	*D. mel*	Std. *vs.* Starvation	0.00312	0.00624	1.34	large
22	wt	*Zim53*	*D. mel*	Rapa *vs.* Starvation	0.104	0.104	−0.668	medium
23	wt	OreR	*D. mel*	Std. *vs.* Rapa	0.00884	0.0265	1.84	large
24	wt	OreR	*D. mel*	Std. *vs.* Starvation	0.0261	0.0521	1.39	large
25	wt	OreR	*D. mel*	Rapa *vs.* Starvation	0.0812	0.0812	−1.09	large
26	wt	sm21	*D. sim*	Std. *vs.* Rapa	0.491	0.52	−0.282	small
27	wt	sm21	*D. sim*	Std. *vs.* Starvation	0.26	0.52	0.466	small
28	wt	sm21	*D. sim*	Rapa *vs.* Starvation	0.039	0.117	0.865	large
29	wt	si1	*D. sim*	Std. *vs.* Rapa	0.148	0.295	0.837	large
30	wt	si1	*D. sim*	Std. *vs.* Starvation	0.00414	0.0124	1.97	large
31	wt	si1	*D. sim*	Rapa *vs.* Starvation	0.352	0.352	0.521	medium
32	Std.		*w1118*	D. mel *vs.* D. sim	0.205	1	0.523	medium
33	Std.		*Sirt4^white+1^*	D. mel *vs.* D. sim	0.0178	0.161	−0.658	medium
34	Rapa		*w1118*	D. mel *vs.* D. sim	0.718	1	0.0948	negligible
35	Rapa		*Sirt4^white+1^*	D. mel *vs.* D. sim	0.971	1	−0.00939	negligible
36	*w^1118^*		*D. mel*	Std. *vs.* Rapa	0.0000251	0.000302	0.93	large
37	*w^1118^*		*D. sim*	Std. *vs.* Rapa	0.242	1	0.369	small
38	*Sirt4^white+1^*		*D. mel*	Std. *vs.* Rapa	0.000432	0.00432	0.765	medium
39	*Sirt4^white+1^*		*D. sim*	Std. *vs.* Rapa	0.000212	0.00234	1.27	large
40	Std.		*D. mel*	*w^1118^ vs. Sirt4^white+1^*	0.0388	0.31	0.438	small
41	Std.		*D. sim*	*w^1118^ vs. Sirt4^white+1^*	0.0406	0.31	−0.675	medium
42	Rapa		*D. mel*	*w^1118^ vs. Sirt4^white+1^*	0.0898	0.539	0.358	small
43	Rapa		*D. sim*	*w^1118^ vs. Sirt4^white+1^*	0.459	1	0.226	small

### Substitution rate of Sirt4

Multiple sequence alignments (MSA) of 183 Sirt4 protein orthologs were performed using ProbCons: Probabilistic consistency-based multiple sequence alignment (Do *et al.* 2005). ProbCons has consistently been evaluated to be more accurate across several alignment benchmarks (BAliBASE, PREFAB and SABmark) compared to other popular msa tools, particularly when alignments have numerous gaps (Pervez *et al.* 2014). At the time of this study, Sirt4 orthologs from 191 species including mammals, fish, birds, and *D. melanogaster* were queried from ensemble.org but only 1:1 orthologs were selected (Useast.ensembl.org1 2018). Additionally, orthologs with missing amino acid sequences were omitted from the MSA. The alignments of the nucleic protein-coding sequences were generated from the protein alignments using the unaligned nucleotide orthologs as guides.

The rate of evolution of *Sirt4* was determined by calculating the ratio of nonsynonymous mutations (d_N_ or K_A_) to synonymous mutations (d_S_ or K_S_). Generally, a K_A_/K_S_ ratio that is significantly above one would indicate a gene that is undergoing a high rate of evolution, and is likely experiencing positive selection. A ratio around one suggests neutral selective pressure on the gene. A ratio profoundly below one characterizes a gene with low genetic drift, which indicates evolutionary conservedness of the protein-coding sequences; this is referred to as negative or purifying selection. For each pairwise alignment, the nonsynonymous (K_A_) and synonymous (K_S_) substitution values were calculated as described (Li 1993):

$$K_{A}=A_{0}+\left(L_{0}*B_{0}+L_{2}*B_{2}\right)/\left(L_{0}+L_{2}\right)$$

$$K_{S}=\left(L_{2}*A_{2}+L_{4}*A_{4}\right)/\left(L_{2}+L_{4}\right)+B_{4}$$

The variables L_0_, L_2_, and L_4_ represent the occurrence of nonsynonymous, synonymous at twofold synonymous, and fourfold synonymous sites respectively; A_0_, A_2_, and A_4_ represent base transition mutations at nonsynonymous, twofold synonymous, and fourfold synonymous sites respectively; B_0_, B_2_, and B_4_ represent base transversions at nonsynonymous, twofold synonymous, and fourfold synonymous sites respectively. Fourfold synonymous sites refers to codons that are translated into amino acids, which can be encoded by four different codons (proline, valine, alanine, and glycine). The same principle applies to twofold synonymous sites. Multiple genome-wide studies across several model organisms and disease states converge toward similar conclusions of base transitions being more prevalent than transversions (Wakeley 1996; Petrov and Hartl 1999; Zhang and Gerstein 2003; Iengar 2012); this is factored into (Li 1993) calculations in which base transition mutations are weighed more heavily for synonymous mutations, whereas transversions contribute more to nonsynonymous mutations. In our analyses, saturated nonsynonymous and synonymous substitutions were coerced to a value of 9.999999.

### Phylogenetic analysis of Sirt4 DNA and protein homologs

*Sirt4* paralogs were downloaded from ensembl.org, and the bacterial sirtuins were downloaded from NCBI (https://www.ncbi.nlm.nih.gov). The sequences were aligned using ProbCons: Probabilistic Consistency-based Multiple Sequence Alignment. The neighbor-joining tree estimation was used to generate the distance matrices, and then the maximum likelihood method was applied to optimize the parameters of the models. The bootstrap analysis was performed across 1000 samples.

### Box and whisker plots

Box and whisker plots depict the spread of variation across populations. The box denotes the interquartile range (IQR) of values between the 25^th^ percentile (Q_1_) and 75^th^ percentile (Q_3_), and the black line within the IQR represents the median (50^th^ percentile). The bottom whisker is calculated as the smallest observation greater than or equal to Q_1_ - 1.5 * IQR, whereas the top whisker is calculated as the largest observation less than or equal to Q_3_ + 1.5 * IQR. The black dots represent outliers, which are values that are between 1.5 and 3 times the IQR. For each figure, the *y*-axis scales oxygen consumption normalized to a reference sample (*OreR w^1118^* in standard treatment).

### R packages

**Figure 2:**
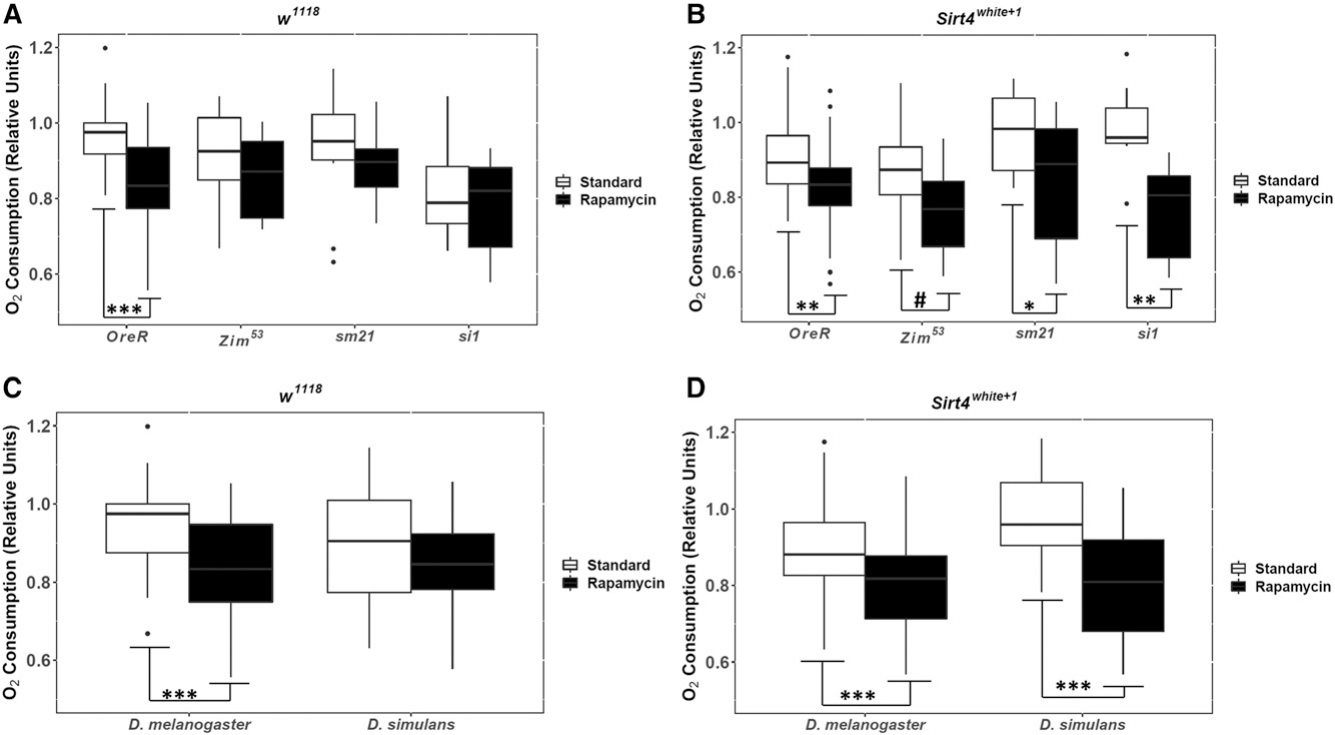
The effects of rapamycin on the oxygen consumption during the presence (*w^1118^*) and absence (*Sirt4white*^*+1*^) of the *Sirt4* gene. (A) and (B) represent the mitochondrial haplotypes grouped by strains. (C) and (D) represent haplotypes grouped by species of the mtDNA. For each pairwise comparison, the raw p-values, Holm-Bonferroni corrected p-values, and Hedges’ g effect sizes are presented in Table 1 and File S2. The significance bars reflect the raw p-values. # = 0.05 < *P* < 0.1; * = 0.01 < *P* < 0.05; ** = 0.001 < *P* < 0.01; *** = *P* < 0.001.

The following R packages and relevant functions were used in the analyses to produce: Figure 1, 2, 3 4: ggplot2::ggplot and ggplot2::geom_boxplot (Wickham 2016). Figure 5: ggplot2::ggplot, ggplot2::geom_boxplot, ggplot2::geom (Wickham 2016) and Rmisc::summarySE (Hope 2013). Figure 6A: ggplot2::ggplot, ggplot2::geom_boxplot (Wickham 2016) and ggplot2::geom_text_repel (Slowikowski 2018). Figure 7: seqinr::kaks, seqinr::reverse.align, seqinr::translate (Charif and Lobry 2007), phylotools::rm.sequence.fasta (Zhang 2017), and graphics::hist (R Core Team 2019). Figure 8: ape::read.dna, ape::read.aa, ape::cophenetic.phylo (Paradis and Schliep 2019), phangorn::phyDat, phangorn::dist.ml, phangorn::NJ, phangorn::pml, phangorn::optim.pml, phangorn::bootstrap.pml, phangorn::plotBS (Schliep 2011; Schliep *et al.* 2017), and ggtree::msaplot (Yu *et al.* 2018). Table 1: car:: Anova, car::leveneTest (Fox *et al.* 2019), stats::shapiro.test, stats::p.adjust (R Core Team 2019), and effsize::cohen.d.formula (Torchiano, 2017). Table 2: car::Anova (Fox *et al.* 2019) and sjstats::anova_stats (Lüdecke 2019). Figure S2A: PerformanceAnalytics::chart.Correlation (Peterson and Carl 2019). Figure S2B: ggplot2::ggplot, ggplot2::geom_boxplot, and ggplot2::geom_dotplot (Wickham 2016).

**Figure 3:**
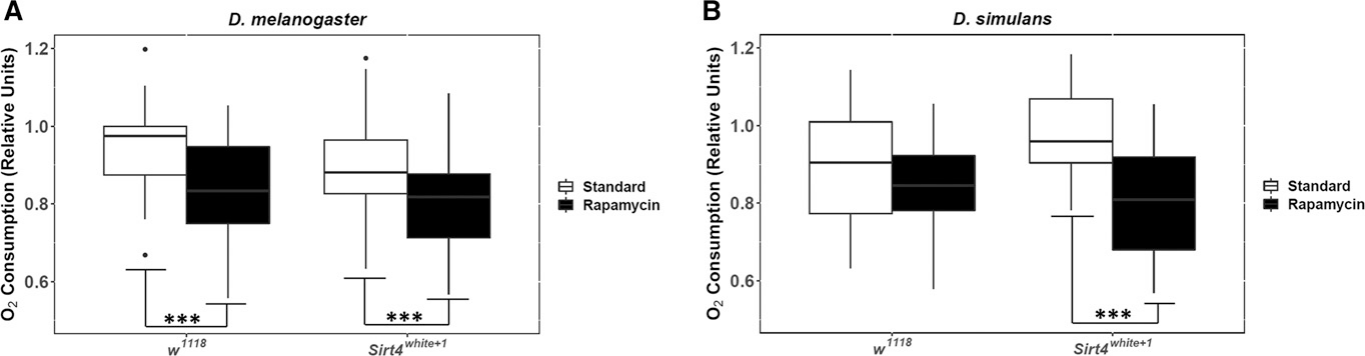
mtDNA species-dependent responses to the deletion of *Sirt4* and rapamycin exposure. Grouped *D. melanogaster* mtDNA (*OreR;OreR* and *Zim^53^;OreR*) is represented in (A) and grouped *D. simulans* introgressed mtDNA (*sm21;OreR* and *sI1;OreR*) in (B). For each pairwise comparison, the raw p-values, Holm-Bonferroni corrected p-values, and Hedges’ g effect sizes are presented in Table 1 and File S2. The significance bars reflect the raw p-values. # = 0.05 < *P* < 0.1; * = 0.01 < *P* < 0.05; ** = 0.001 < *P* < 0.01; *** = *P* < 0.001.

**Figure 4:**
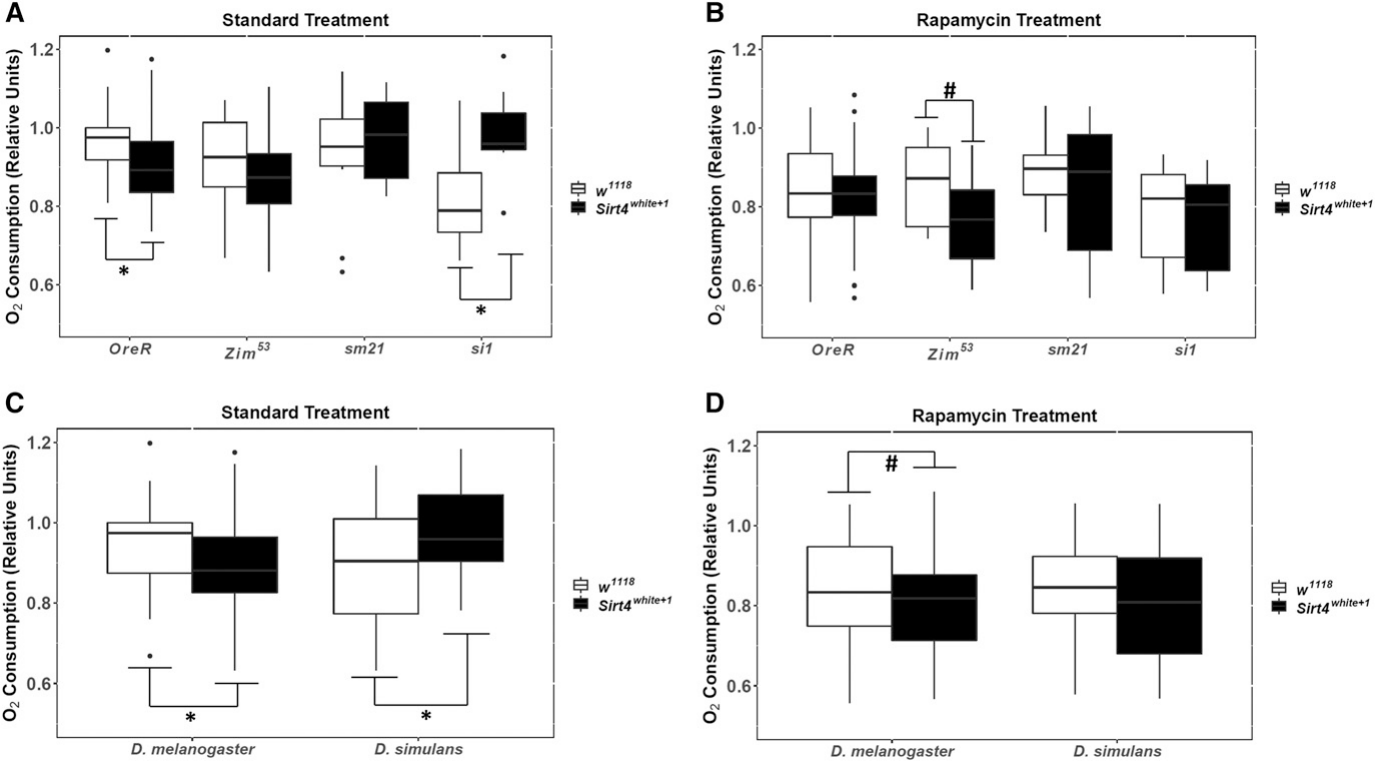
The effects of *Sirt4* deletion on oxygen consumption among the standard and rapamycin treatments. Mitochondrial haplotypes grouped by strains are represented in (A) and (B). mtDNA haplotypes grouped by species are represented in (C) and (D). For each pairwise comparison, the raw p-values, Holm-Bonferroni corrected p-values, and Hedges’ g effect sizes are presented in Table 1 and File S2. The significance bars reflect the raw p-values. # = 0.05 < *P* < 0.1; * = 0.01 < *P* < 0.05; ** = 0.001 < *P* < 0.01; *** = *P* < 0.001.

**Figure 5:**
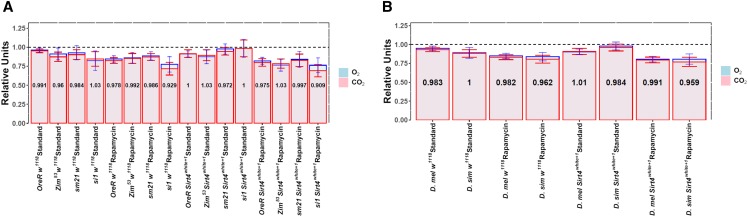
Comparison of the effects of rapamycin on the consumption of oxygen (blue) and the production of carbon dioxide (pink) during the presence (*w^1118^*) and absence (*Sirt4white*^*+1*^) of the *Sirt4* gene. (A) Mitochondrial haplotypes grouped by strains. (B) Haplotypes grouped by species of mtDNA. Error bars represent the 95% confidence interval. The number inside the bars represents the mean respiratory quotient (RQ): volume of CO_2_/volume of O_2_. All values were normalized to the reference sample (*OreR w^1118^* in standard treatment).

**Figure 6:**
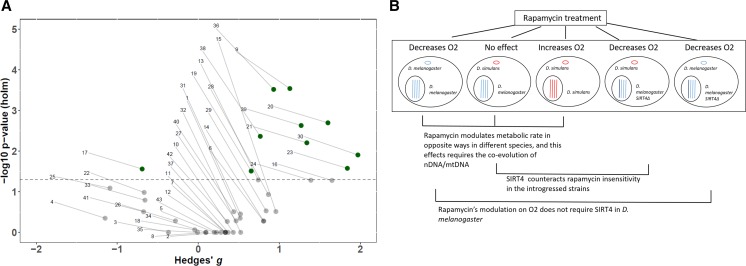
(A) Volcano plot depicting the spread of all of the pair-wise analyses. The y-axis scales the p-values (log10-transformed) that were adjusted for multiple comparisons using the Holm-Bonferroni method, and the x-axis scales the Hedges’ g effect sizes for each pair-wise comparison. The dotted line represents the alpha level of 0.05; green samples above the line are significant (*P* < 0.05), whereas the gray samples below the line are not significant (*P* > 0.05). The numbers pointing to each dot corresponds with the ID # on Table 1 and File S2. (B) Graphical representation summarizing the impact that rapamycin treatment has on modulating oxygen consumption for each condition used in this research. Each big circle represents a distinct cell that corresponds with a strain used in this experiment. The small circle within the cell represents the species of the mtDNA. The lines within the black circle (nucleus) represent the nuclear chromosomes. Blue represents DNA from *D. melanogaster* and red corresponds with *D. simulans* DNA. The deletion of *Sirt4* is labeled as a darker blue chromosome.

**Figure 7:**
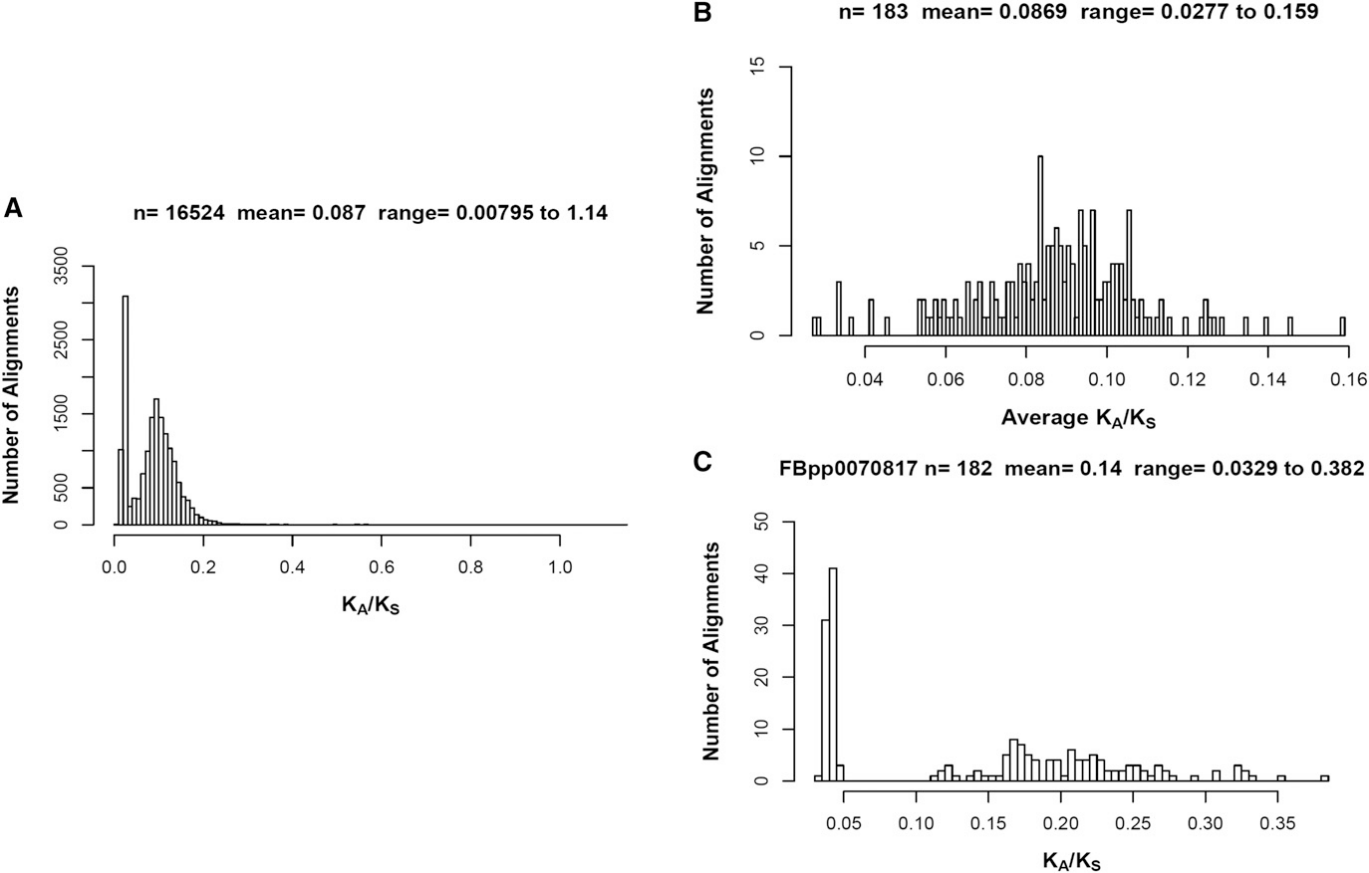
Multiple sequence alignments of 183 *Sirt4* orthologs. (A) Histogram of the K_A_/K_S_ substitution ratio for all of the pairwise alignments. (B) The average K_A_/K_S_ from each species was plotted as a histogram. (C) Histogram plot of the K_A_/K_S_ ratio from all of pairwise alignments with *D. melanogaster* (Ensembl Peptide ID: FBpp0070817).

**Figure 8:**
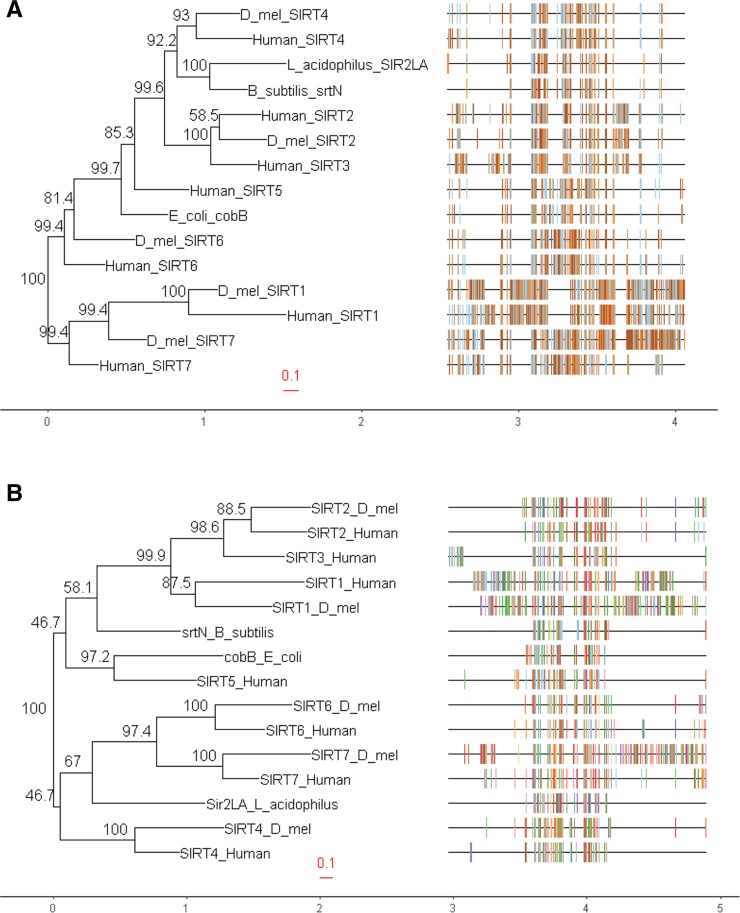
Phylogeny of human and *D. melanogaster **Sirt4* paralogs and proteobacterial Sirtuins. (A) Phylogeny generated from DNA sequences. (B) Phylogeny generated from protein sequences. At each node, the numbers represent the bootstrap support values as a percentage; the higher the value, the more reliable the grouping at the node. The multiple sequence alignments are displayed to the right of the phylogeny.

**Table 2:** ANOVA was performed on a model in which oxygen consumption normalized to the reference sample (*OreR w^1118^* under standard treatment) was the dependent variable, and the independent variables were the following: genotype (presence or deletion of *Sirt4*), treatment (standard or rapamycin) and mtDNA (*D. melanogaster* or *D. simulans*). Type 3 ANOVA was selected for the omnibus analysis due to the model containing significant interaction terms. P-values and various effect size estimates were computed to reflect the variance that can be explained by each explanatory variable on the normalized oxygen consumption. The traditional magnitude conventions accompany relevant effect size values: L= large effect; M= medium effect; S= small effect (Cohen 1988; Kirk 1996; Kotrlik and Williams 2003)

term	sumsq	meansq	df	statistic	p.value	etasq	partial.etasq	omegasq	partial.omegasq	cohens.f	power
(Intercept)	170.18961	170.18961	1	11614.34465	0	0.97433	0.97876	0.97416	0.97802	6.78886	1
Genotype	0.00371	0.00371	1	0.25317	0.61529	0.00002	0.001	−0.00006	−0.00287	0.0317	0.07948
mtDNA	0.00024	0.00024	1	0.01623	0.89874	0	0.00006	−0.00008	−0.00378	0.00802	0.05186
Treatment	0.60305	0.60305	1	41.15459	0	0.00345	0.14039	0.00337	0.13334	0.40412	1
Genotype:mtDNA	0.07883	0.07883	1	5.3799	0.02117	0.00045	0.0209	0.00037	0.0165	0.14611	0.64051
Genotype:Treatment	0.04789	0.04789	1	3.26796	0.07184	0.00027	0.0128	0.00019	0.00861	0.11388	0.43969
mtDNA:Treatment	0.00275	0.00275	1	0.18779	0.66513	0.00002	0.00074	−0.00007	−0.00312	0.0273	0.07178
Genotype:mtDNA:Treatment	0.05487	0.05487	1	3.74434	0.05411	0.00031	0.01464	0.00023	0.01041	0.1219	0.49021
Residuals	3.69266	0.01465	252								

### Data availability

The *Drosophila* strains are available upon request. Figure S1 shows PCR confirmation of the *w^1118^* and *Sirt4white*^*+1*^ strains. Figure S2A presents a correlation matrix and Figure S2B is a boxplot on the relationship between treatment and fly movement. File S1 contains all data for the normalized O_2_ consumption, CO_2_ production, and RQ values that were used to generate the figures. File S2 contains the statistics for all analyses related to O_2_ consumption. Supplemental material available at figshare: https://doi.org/10.25387/g3.11888910.

## Results

### Rapamycin experts oppositemetabolic effectson coevolved mtDNA/nDNA and introgressed strains

To study the effect of rapamycin on the metabolism of living organisms we measured oxygen consumption as a metric for routine metabolic rate, given that oxygen is the final electron acceptor during aerobic respiration. Rapamycin treatment on wildtype *D. melanogaster* decreased the consumption of oxygen when compared to the standard treatment (*P* < 0.01 and *P* < 0.001 for *OreR;OreR* and *Zim^53^;OreR* respectively), which was a trend that was also present under starvation (*P* < 0.05 and *P* < 0.01 for *OreR;OreR* and *Zim^53^;OreR* respectively) (Figure 1B-C and Table 1). Surprisingly, an opposite effect was seen in *C167.4;C167.4* (wildtype *D. simulans*) in which rapamycin treatment increased the consumption of oxygen (*P* < 0.01), but starvation had no effect (*P* = 0.866) (Figure 1D and Table 1). However, the rate of oxygen consumption among the introgressed strains was not affected by rapamycin treatment (*P* = 0.491 and *P* = 0.148 for *sm21;OreR* and *si1;OreR* respectively), which further supports the role of coevolved mtDNA and nDNA in regulating metabolic rate in whole-flies (Figure 1E-F and Table 1).

### Sirt4 modulates response to rapamycin in a species-dependent manner

In this experiment, we examined the role of Sirt4 in the response to the rapamycin-mediated modulation of oxygen consumption, which served as a proxy for metabolic rate. ANOVA was conducted to reveal the impact of the following variables on oxygen consumption normalized to the reference sample (*OreR w^1118^* in standard treatment): genotype (presence or absence of *Sirt4*), treatment (ethanol vehicle or rapamycin), and mtDNA (*D. melanogaster* or *D. simulans*). The following had significant effects on whole-fly oxygen consumption: treatment as a main predictor (*P* < 0.0005) and genotype:mtDNA as an interaction (*P* < 0.05) (Table 2). Genotype:treatment:mtDNA had a borderline significant (*P* = 0.0541) impact on oxygen consumption (Table 2). Effect sizes were calculated in order to determine the magnitude of effect that each explanatory variable had on the normalized oxygen consumption. Within this study, the omega-squared value (ω^2^) was selected to compare the influence of each predictor on oxygen consumption, considering that ω^2^ is less biased against population variances compared to eta-squared (Lakens 2013). Accordingly, treatment had the greatest influence on oxygen consumption (ω^2^ = 0.0034), whereas genotype:mtDNA (ω^2^ = 0.0004) and genotype:treatment:mtDNA (ω^2^ = 0.0002) had relatively marginal impacts on oxygen consumption (Table 2). An important consideration is that effect size calculations can be heavily masked in omnibus analyses with many explanatory variables (seven in this experiment). To correct for this, the partial ω^2^ effect sizes were also reported for between-studies applications.

The *Drosophila* strains demonstrated distinct trends in oxygen consumption under rapamycin treatment as well as the expression or deletion of *Sirt4* (Figure 2A-B). Our data suggests that these trends were driven by the cumulative interplay between treatment, expression/deletion of *Sirt4*, and the species of the mtDNA haplotypes (Table 2). In addition, preliminary statistics favored mtDNA as a grouping variable as opposed to individual strains, indicating that *D. melanogaster* mtDNA haplotypes (*OreR;OreR* and *Zim^53^;OreR*) displayed similar metabolic trends under all conditions, whereas the *D. simulans* mtDNA haplotypes (*siI;OreR* and *sm21;OreR*) shared similar trends in oxygen consumption (Figure 2A-B, and Figure 4A-B). For this reason, the majority of the data were analyzed and graphically presented based on the species of the mtDNA haplotype (hereafter identified as either *D. melanogaster* or *D. simulans*).

The routine metabolic rates of *w^1118^* in standard and rapamycin treatments were statistically similar to the progenitor strains that were transgenically manipulated through ends-out recombination (data not shown). As such, *w^1118^* was considered the genotypic control in this experiment. Under standard treatment, the trends in oxygen consumption exhibited by *w^1118^* reflected similar metabolic rates between *D. simulans* introgressed lines and *D. melanogaster* (*P* = 0.205) (Figure 2C and Table 1). Conversely. *D. simulans* experienced higher oxygen consumption than *D. melanogaster* (*P* < 0.05) when *Sirt4* is deleted (Figure 2D and Table 1). However, the observed differences in oxygen consumption between the mtDNA haplotypes were eliminated under rapamycin treatment (Figure 2D and Table 1).

*D. melanogaster w^1118^* exhibited significantly lower oxygen consumption when administered rapamycin (*P* < 0.001) (Figure 3A and Table 1), whereas *D. simulans w^1118^* were metabolically unresponsive to rapamycin (Figure 3B and Table 1). This data are consistent with results shown in Figure 1 that characterized changes in metabolic rate exhibited by each mtDNA haplotype in response to rapamycin. Similarly, these results are corroborated by data published by Villa-Cuesta *et al.* (2014) who demonstrated that isolated mitochondria from introgressed lines had stagnant oxygen consumption when fed rapamycin. However, *D. simulans Sirt4white*^*+1*^ were highly sensitive to rapamycin (*P* < 0.01) with the greatest observed reduction in oxygen consumption (Hedges’ g = 1.27) among the mtDNA analyses (Figure 3B, Figure 6A, and Table 1). These results strongly implicate Sirt4 acts as an antagonist/inhibitor of the rapamycin-mediated reduction in oxygen consumption among *D. simulans* introgressed lines.

Interestingly, *D. melanogaster Sirt4white*^*+1*^ still exhibited reduced oxygen consumption under rapamycin treatment, although the magnitude of effect (Hedges’ g = 0.765) (Figure 6A) was smaller compared to *D. melanogaster w^1118^* administered rapamycin (Hedges’ g = 0.93) (Figure 3A and Table 1). This may indicate that rapamycin modulates *D. melanogaster* oxygen consumption through Sirt4-independent pathways. Even so, it appears that Sirt4 has a marginal/subtle influence on amplifying rapamycin’s mode of action in *D. melanogaster*.

### Sirt4 deletion has species-dependent effects on oxygen consumption

The deletion of *Sirt4* decreased *D. melanogaster* oxygen consumption (*P* < 0.05), whereas oxygen consumption was increased among *D. simulans* introgressed strains (*P* < 0.05) under standard treatment (Figure 4C and Table 1). However, the species-dependent effects of Sirt4 was largely reduced under rapamycin treatment (Figure 4D), indicating that rapamycin exposure in conjunction with the deletion of *Sirt4* did not result in an additive influence on oxygen consumption in *D. melanogaster*.

### Sirt4 deletion does not alter respiratory quotients

To determine differences between the catabolism of macronutrients in strains of flies treated with rapamycin in the presence or absence of Sirt4, we calculated the respiratory quotient (RQ): the ratio between carbon dioxide production and the consumption of O_2_. The mean Vo_2_ and Vco_2_ were similarly affected by rapamycin treatment across all strains (Figure 5A). Accordingly, our data show negligible differences in the mean RQ among our samples (*P* = 0.32; ω^2^ = 0; partial ω^2^=-0.00009) (Figure 5). In addition, there was a strong correlation (R = 0.9; *P* < 0.01) between the normalized O_2_ and CO_2_ values, whereas the RQ values weakly correlated with O_2_ consumption (R=-0.28; *P* < 0.001) and CO_2_ production (R = 0.16; *P* < 0.01) (Figure S2A). Therefore, the interaction between *Sirt4* deletion, rapamycin treatment, and different mtDNAs does not significantly shift the source of fuel used by the strains.

### Sirt4 exhibits purifying selection across 183 species

Since there is not much information on the genetic drift of *Sirt4*, we calculated the *Sirt4* rate of evolution by analysis of substitution (K_A_/K_S_) ratios on 183 *Sirt4* orthologs from ensembl.org (Useast.ensembl.org1. 2018). We found that the average substitution rate was 0.087 (Figure 7A). The highest rate of evolution (K_A_/K_S_ = 1.14) was between *Apteryx rowi* and *Apteryx haastii*, and the lowest substitution rate (K_A_/K_S_ of 0.00795) was between *Meleagris gallopavo* and *Crocodylus porosus* (Figure 7A). The species with the lowest average substitution rate (average K_A_/K_S_ of 0.0277) was *Eptatretus burgeri*, whereas *Kryptolebias marmoratus* exhibited the most genetic drift (average K_A_/K_S_ of 0.159) (Figure 7B). Among all pairwise alignments involving *D. melanogaster*, the average K_A_/K_S_ was 0.154; the lowest substitution rate (K_A_/K_S_ = 0.0329) was with *Notechis scutatus*, whereas the highest substitution rate (K_A_/K_S_ = 0.382) was with *Fundulus heteroclitus* (Figure 7C). Overall, *Sirt4* exhibits a high degree of purifying selection across very divergent species.

## Discussion

Nutrient-sensing pathways are fundamental in maintaining metabolic and cellular homeostasis. Cytosolic metabolic pathways, such as TOR, streamline communication between the mitochondria and nucleus in response to shifts in metabolites and energy demands. However, effective nuclear-mitochondrial crosstalk is also facilitated through the activities of effector proteins localized throughout the mitochondria. Perturbing the coevolution between the mitochondrial and nuclear genomes similarly disrupts the crosstalk between the mitochondria and nucleus, which could have downstream effects on metabolism.

Our research shows an opposite effect on the metabolic rate of *D. melanogaster* and *D. simulans* upon the inhibition of TOR by the drug rapamycin. Furthermore, we present data that links rapamycin’s activity, in altering mitochondrial metabolism, with the coevolution between mtDNA and nDNA. This is evident as the introgression of mtDNA from *D. simulans* into a fly harboring *D. melanogaster* nDNA abolishes the responsiveness to rapamycin treatment (Figure 1B-F). While rapamycin has been shown to modify metabolic rate and mitochondrial efficiency through mtDNA-dependent reprogramming of mitochondrial metabolism (Villa-Cuesta *et al.* 2014), the mode as to how rapamycin is regulating these effects might lay at the core of the differential activity of Sirt4 under altered mitochondrial-nuclear epistatic cues (summarized in Figure 6B).

Although our research does not provide the direct mechanism as to how rapamycin treatment is affecting metabolic rate, we show that the oxygen consumption of *D. melanogaster* (coevolved nuclear and mitochondrial genomes) is marginally influenced by the activity of Sirt4 (Figure 3A). This strongly suggests that rapamycin’s mechanistic activity in *D. melanogaster* is not fully conferred through Sirt4-dependent pathways. Even so, effect size comparisons of the response to rapamycin between *w^1118^* (Hedges’ g = 0.93) and *Sirt4white*^*+1*^ (Hedges’ g = 0.765) indicate that Sirt4 may subtly enhance rapamycin’s metabolic effects (Figure 6A). While it is apparent that Sirt4 and rapamycin’s metabolic roles are decoupled in strains with coevolved nDNA and mtDNA, our results show that Sirt4 is necessary for the resistance to rapamycin in introgressed lines. In particular, our results indicate that Sirt4 potentially operates as an inhibitor of rapamycin-mediated reduction in oxygen consumption when the mitochondrial and nuclear genomes are not coevolved (Figure 3B and 5B). It is uncertain whether this Sirt4 phenotype is conferred through inhibition of targets of rapamycin, or if Sirt4-dependent pathways upregulate metabolic rate through separate compensatory mechanisms which may ostensibly counteract the rapamycin-mediated reduction in oxygen consumption. In both cases, it is clear that when the coevolution between the nuclear and mitochondrial genomes is disrupted, Sirt4 adopts a new role in metabolism that intersects with and antagonizes rapamycin’s effect on mitochondrial metabolism. Considering that rapamycin inhibits TOR signaling, our data suggest that Sirt4 is differentially regulated by TOR between the introgressed and coevolved *D. melanogaster* strains. As an explanation to our results, we propose that a disruption in the coevolution between the nDNA and mtDNA remodels Sirt4-dependent pathways in a novel interaction with TOR that results in new physiological responses. In particular, Sirt4 appears to behave as a negative feedback regulator of mitochondrial metabolism in introgressed strains, whereas Sirt4 functions as an agonist of mitochondrial metabolism in coevolved *D. melanogaster* mtDNA haplotypes (Figure 4C). However, the metabolic responses to the deletion of *Sirt4* are eliminated under rapamycin treatment (Figure 4D). This feature may indicate that the extent of reducing oxygen consumption, induced by metabolic pathways regulated by Sirt4 and/or rapamycin, is limited past a certain threshold or metabolic set-point.

It is worth mentioning that although we measured routine metabolic rate by volume of oxygen consumed, the production of carbon dioxide was similarly affected by rapamycin treatment across all strains (Figure 5). We also calculated the respiratory quotient (RQ) to determine if there were differences in the primary macronutrients (fats, proteins, carbohydrates) metabolized by the flies. Our analyses do not reveal meaningful differences in RQ among our samples (Figure 5) suggesting that the deletion of *Sirt4*, in conjunction with rapamycin exposure, does not modify the source of fuel used by the mtDNA haplotypes. More thorough metabolomics studies may yield valuable information into the shifts of specific metabolites when *Sirt4* is deleted.

Interestingly, Sirt4 research in mammalian organisms converge toward an inhibitory/antagonistic regulation of metabolism, but recent studies in *Drosophila* suggest that Sirt4 acts as an agonist of mitochondrial anaplerosis conferring enhanced metabolism of energy stores such as fatty acids, glucose, intermediates in the Krebs cycle, and amino acid metabolites (Wood *et al.* 2018). There are currently no theories to explain Sirt4’s evolutionary pleiotropy. However, sequence alignments of 183 *Sirt4* orthologs reveal an average nonsynonymous/synonymous (K_A_/K_S_) substitution ratio of about 0.1, suggesting that *Sirt4* is undergoing strong purifying selection with minimal genetic drift (Figure 7). In addition, our research is the first to present evidence of disparate *in vivo* Sirt4 metabolic phenotypes among evolutionarily divergent *Drosophila* mtDNA haplotypes (Figure 4C), indicating that the pleiotropy of Sirt4’s effect on metabolism is not a feature that is exclusive to extremely divergent phylogenetic orderings of organisms, but is also expressed among closely related species within a genus.

As a key regulator of metabolism, Sirt4 exhibits a diverse range of enzymatic activities including inhibition of GLUD1 through ADP-ribosylation (Haigis *et al.* 2006), downregulation of mitochondrial genes that promote fatty acid β-oxidation (Nasrin *et al.* 2010), and lipoamidase cleavage of the DLAT complex of pyruvate dehydrogenase (Mathias *et al.* 2014). Therefore, a differential regulation of lipids and or amino acid metabolisms may provide species contrast and a channel for synchronizing metabolic needs between mitochondria and the rest of the cell. Taking all in consideration, we propose that Sirt4 differentially coordinates metabolic rate and responsiveness to rapamycin across mtDNA haplotypes, which implicates Sirt4 as a conditional modulator of TOR-mediated signaling pathways.

Another factor to consider is that species-dependent mRNA splicing may dictate the production of *Sirt4* transcript variants across different mtDNA haplotypes. In *D. melanogaster*, at least three protein-coding transcripts have been identified: *FBtr0070850* (*Sirt4-RA*), *FBtr0070851* (*Sirt4-RB*), and *FBtr0070852* (*Sirt4-RC*) (Useast.ensembl.org2 2018). The *Sirt4-RA* and *Sirt4-RB* transcripts are 229 residues and have similar proteomic features but differ in that *Sirt4-RA* has 3 exons and *Sirt4-RB* has 2 exons. On the other hand, *Sirt4-RC* displays the following notable differences in structure: 312 residues, slightly more basic residues, and a HAMAP domain belonging to the Sirtuin Class II family (Useast.ensembl.org3 2018). In addition, several sites crucial in Sirt4 chelation of zinc are variably present among the transcripts. Since only the *Sirt4-RC* translated peptide has been characterized, there is a need for further research to elucidate the phenotypes of *Sirt4-RA* and *Sirt4-RB*. Therefore, changes in the expression of *Sirt4* transcripts, and possibly the function of Sirt4, may be induced under unique mitochondrial-nuclear epistatic interactions that are perturbed in strains with non-coevolved nuclear and mitochondrial genomes. While the mechanisms of retrograde signaling are largely unclear, states of metabolic stress and aberrations in the mitochondrial genome might instigate these signaling pathways. Transcription factors, such as GPS2, NFκB, and Rtg2, are pivotal in retrograde signaling and concomitant regulation of nuclear genes encoding mitochondrial proteins in response to perturbations in cellular metabolism, cell stress, reactive oxygen species, or mitochondrial dysfunction (Finley and Haigis 2009; Jazwinski and Kriete 2012; Cardamone *et al.* 2018). In our research, the coevolution between the nDNA and mtDNA is disrupted in introgressed lines, which the cell may perceive as an altered or dysfunctional mitochondrial state thereby activating retrograde signaling pathways. Through these mechanisms, introgressed strains may have variable expressions of *Sirt4* transcript variants, or altered post-translational regulation of Sirt4, which in turn could produce unique physiological responses. This may account for the intrinsic differences in Sirt4 phenotypes between the mtDNA haplotypes as well as the species-dependent responses to rapamycin.

Lastly, sirtuins have been discovered in several microbial organisms. Among these proteobacterial sirtuins, SirTMs have been characterized as ADP-ribosyltransferases in pathogenic bacteria (Rack *et al.* 2015), Sir2La hydrolyzes short chain ε-N-acyllysine in *Lactobacillus acidophilus* (Olesen *et al.* 2018), and cobB deacetylates acetyl-CoA synthetase in *Escherichia coli* (Zhao *et al.* 2004). Additionally, cobB and srtN have been identified as functional lipoamidases through catalytic interactions with pyruvate dehydrogenase (a known target of Sirt4) and alpha-ketoglutarate dehydrogenase (Rowland *et al.* 2017). However, *Sirt4* orthologs have not been recognized in single-cell organisms, and simple fungal blast searches of Sirt4 protein sequences yield poor results besides homology with sir2 sequences. Based on all this information, we theorize that a progenitor *Sirtuin* gene, from a proto-mitochondria, was integrated into the nDNA, and through iterative mutations and other selective pressures, *Sirt4* evolved as an extant nuclear gene - a gene that expresses an enzyme localized to the mitochondria where it functions as a master regulator of oxidative metabolism. This theory is supported by our phylogenetic tree which shows that human and *D. melanogaster **Sirt4* DNA sequences are more closely related with the proteobacterial Sirtuins (*srtN* and *cobB*) and *Sirt5* compared to other human and fruit fly *Sirt4* paralogs (Figure 8A). More interestingly, Sirt4 protein sequences share the closest common ancestors with srtN, Sirt5, Sir2La, and cobB (Figure 8B).

The absence of *Sirt4* in microbial organisms, combined with the low evolutionary rate in eukaryotes (Figure 7), strongly indicates selection for the gene, likely in order to meet the demands for regulating metabolic processes that are key in higher-order organisms but periphery in microbes. Accordingly, increasing diversity in dietary sources may have selected for similarly diverse roles of Sirt4 in regulating the anapleurtic proccesses of the Krebs Cycle. We propose that the localization of Sirt4 fosters obsequiousness to the crosstalk between the mitochondria and other cellular compartments that is ultimately governed by an intricate network of mitochondrial-cellular epistatic cues.

In sum, our results show that Sirt4 regulates metabolic homeostasis through disparate phenotypes between *D. melanogaster* and *D. simulans* mtDNA haplotypes. Our research provides evidence that metabolism is not solely regulated by the maintenance of substrates or the bioavailability of nutrients, but also intrinsic conditions such as the optimal interactions of genes encoded by the mitochondrial and nuclear genomes. Given that our data indicates that Sirt4 abrogates rapamycin activity in introgressed strains, but not in *D. melanogaster*, it is possible that rapamycin’s therapeutic effect can be modulated through Sirt4 under irregular mitochondrial states. Furthermore, considering that Sirt4 functions as a negative regulator of oxidative metabolism in humans and in *D. simulans* mtDNA haplotypes (Figure 3A-B), *D. simulans* may be a more appropriate model organism compared to *D. melanogaster* for research on Sirt4 phenotypes and Sirt4-related metabolic disorders. Further investigations may enhance therapeutic interventions for patients who are unresponsive to rapamycin treatments. As a whole, elucidating the species-dependent mechanisms of Sirt4 phenotypes may shed light onto retrograde signaling and other higher-order epigenetics that govern mitochondrial metabolism.
